# Artificial intelligence in oncology, its scope and future prospects with specific reference to radiation oncology

**DOI:** 10.1259/bjro.20180031

**Published:** 2019-05-13

**Authors:** Rajit Rattan, Tejinder Kataria, Susovan Banerjee, Shikha Goyal, Deepak Gupta, Akshi Pandita, Shyam Bisht, Kushal Narang, Saumya Ranjan Mishra

**Affiliations:** 1 Division of Radiation Oncology, Medanta- The Medicity, Gurgaon, Haryana, India; 2 Department of Dermatology, P. N. Behl Skin Institute, New Delhi, India

## Abstract

**Objective:**

Artificial intelligence (AI) seems to be bridging the gap between the acquisition of data and its meaningful interpretation. These approaches, have shown outstanding capabilities, outperforming most classification and regression methods to date and the ability to automatically learn the most suitable data representation for the task at hand and present it for better correlation. This article tries to sensitize the practising radiation oncologists to understand where the potential role of AI lies and what further can be achieved with it.

**Methods and materials:**

Contemporary literature was searched and the available literature was sorted and an attempt at writing a comprehensive non-systematic review was made.

**Results:**

The article addresses various areas in oncology, especially in the field of radiation oncology, where the work based on AI has been done. Whether it’s the screening modalities, or diagnosis or the prognostic assays, AI has come with more accurately defining results and survival of patients. Various steps and protocols in radiation oncology are now using AI-based methods, like in the steps of planning, segmentation and delivery of radiation. Benefit of AI across all the platforms of health sector may lead to a more refined and personalized medicine in near future.

**Conclusion:**

AI with the use of machine learning and artificial neural networks has come up with faster and more accurate solutions for the problems faced by oncologist. The uses of AI,are likely to get increased exponentially . However, concerns regarding demographic discrepancies in relation to patients, disease and their natural history and reports of manipulation of AI, the ultimate responsibility will rest on the treating physicians.

## Introduction

Radiation Oncology in last 50 years has seen a rapid transformation from clinic and hypothesis-based medical art to technology and evidence-driven science. Every effort has been made to determine the optimum ways of treatment, the required doses, the time frame of delivery, the expected outcome and the scope of improvement. With the introduction of three-dimensional computer planning, we now have, in our hands, various clinical and patient related data that can be interpreted meaningfully, for guidance for future patient care and optimizing further treatment. There has been a rightful dependence, on medical data, that is now being interpreted with increased accuracy and shaped into working knowledge. Artificial intelligence (AI) or machine learning seems to be bridging the gap between the acquisition of data and their meaningful interpretation into oncology. These approaches, have shown outstanding capabilities, outperforming most classification and regression methods to date and the ability to automatically learn the most suitable data representation for the task at hand and present it for better correlation and understanding. This article tries to avoid complex methmatical formulas and derivations and tries to make it simple for the clinicians and researchers to understand how the concept of AI and its various methods like machine or deep learning, convolutional neural network (CNN) are currently being utilized in the field of oncology, what has been achieved till now, and what further can be done in this regard. The authors have kept in mind that all the recent papers that talk about machine learning or AI use in oncology are limited in their ability to make the oncologists understand, as to where the process is currently placed and headed in future.

## methods and materials

Contemporary literature was searched and the available literature was sorted and an attempt at writing a comprehensive non-systematic review was made. Uploads and links related to the use of AI in oncology on various social media sites especially, from twitter handles of various researchers and oncologists are followed by the first author and he formulated the article from his read articles. Key words that were searched were; Artificial intelligence, machine learning, deep learning, convolutional neural networks (CNN), radiation oncology, planning and mammography. Relevant publications were read and analysed and a comprehensive non-systematic review was made.

## Results—Use of AI in Oncology and Radiation Oncology

### Role in screening

Screening methods have long employed the utilization of various risk stratification methods to identify patient populations in which a said non-expensive, easy to interpret method can detect cancer at an early stage. The basic problem with these methods is that they utilize only a limited proportion of patients’ characteristics for risk stratification. For example, screening mammography done to detect breast carcinoma in patients uses only age group of more than 40 years as the parameter of inclusion criteria. There has been conflicting literature regarding its benefit in females, with the data showing no benefit in reducing mortality in patients.^1^ Similar problems have been seen with screening for carcinoma of lung,^2^ prostate^3^ and ovary.^4^ As modalities of investigations, the screening procedures have excellent sensitivity, however, where these tests lack is the fact that a large number of patients have to be screened in order to get those minority of cases which have a detectable cancer in their bodies, thus having low yield and less impact on population.

AI can be of great help in substantially decreasing these number of subject populations to be screened. By analyzing the characteristics of the patients taking in consideration many more factors than what a classical risk stratification programme utilises, it will help categorize population into low- and high-risk cohorts, and screening methods can be instituted accordingly. For example, patients to be screened for breast cancer can be risk stratified based on their age, family, menstrual, smoking history, body mass index and other host of risk factors to determine the probability whether mammography will be helpful in the patient or not. Similar approach has shown results in England, where analyzing patient data alone with machine learning, determined the risk of future cardiac events, superseding all the present probabilistic models^5^ Thus, in future, with the help of AI, cancer screening methods will be expected to be quicker, more precise, cost effective and result yielding.

### Role in diagnosing; emphasis on radiology

AI algorithms, particularly deep learning methods, CNNs and variational auto-encoders, have shown great promise in identifying gross as well as subtle variation in routine imaging. Traditionally, in radiology, trained physicians visually assess the medical images for the detection, characterization and monitoring of diseases. AI methods enable automatic recognition of complex patterns in imaging data, providing quantitative, as well as qualitative assessments of radiographic characteristics within a short period of time. For most conditions, accurate and early diagnosis helps to start treatment earlier with the aim of reducing morbidity, mortality and treatment or disease-related complications. For example, females between 50 and 70 are advised to have mammograms every 3 years to screen for breast cancer.^6^ A high proportion of mammograms has been a major problem yielding false-positive results when interpreted by radiologists.^7^ This leads to about 50% of the healthy being subject to further procedures to rule out breast cancer.^8^ With the help of AI, interpretation of mammograms has become 30 times faster than humans and with greater accuracy.^9^ Lei Zhen^10^ and associates presented an algorithm that combined several artificial intelligent techniques with the discrete wavelet transform (DWT) for detection of masses in mammograms. The verification results showed that the proposed algorithm had a sensitivity of 97.3% and the number of false positives per image were 3.92. This figure is very less when compared with the cumulative false positivity with conventionally screened mammograms which in one review stands at 49% after 10 mammograms.^11^ These false positive tests not only lead to increased healthcare expenditure but also increased patient distress and anxiety level.^12^

Using AI in imaging can tell the radiologist about the suspicious scans that need to be looked at first among the huge number of other normal imaging findings. This has a tremendous potential in reducing time, aiding early diagnosis from the time of the mammogram, reducing the need for unnecessary biopsies and the concern of a misdiagnosis.^13^ Similar techniques to those described above are being deployed for the evaluation of eye imaging, skin lesions, electrocardiograms, X-rays and cross-sectional imaging such as CT or MRI. Use of AI can also aid in complex imaging where lesion characteristics can be non-defining, like classification of pulmonary nodules into benign or malignant..^14^

### Role in prognostication

Cancers are a unique set of diseases in the sense that they are associated with the risk of harbouring micrometastases which lead to increased risk of recurrence, whether local or systemic. Since a long time, cancer management strategies have employed various risk assessment tools for determining the probability of future metastatic potential. For example, characteristics of post-operative histopathology characters like grade, size, local infiltration status, number of lymph nodes involved etc. have been widely used as a marker of recurrence in post-operative cases. In other cases, *e.g.* in prostate cancer, use of nomograms is widespread and possibly one of the earliest ways of deriving risks of locoregional and metastatic recurrence based on experiences in similar clinical situations.^15,16^ This prognostic assessment, has potentiated the emergence of various genetic, molecular markers and signatures in the tumours.^17,18^ However, as shown by the recent results of TailorX,^19^ and the its limited adoption among clinicians^20^ in breast carcinoma, the risk assessment tools are still evolving. Besides this, the conventional risk assessment and treatment tailoring tools use only a limited set of characteristics like stage, histopathology features to access risk of local and systemic recurrence and eventually the indication of adjuvant therapy. AI has the potential of not only utilising the historical parameters like stage, histopathology characteristics, genetic make-up of the tumour, but also has the ability of taking into consideration the different characteristics of a patient like age, sex, performance status, geographical inconsistencies and the historical backgrounds of the disease to formulate meaningful prognostication assays and thus guiding therapy.^21^ By meaningfully analyzing the data, AI generated algorithms can give a more precise estimate of the risk of future locoregional or metastatic potential. Much has already been done in this regard.^22,23^ The precise risk estimation will go a long way in ensuring proper tailored treatment on an individual basis and further follow-up actions can be made more result oriented and cost effective.

### AI in radiation oncology

Patient treatment workflow in radiotherapy includes many steps like patient positioning and immobilization, acquisition of planning CT, segmentation of tumour and organs at risk (OARs), radiation planning and determining the preferred dose, time and fractionation schedule, optimizing the beam positions for optimum dose coverage and normal tissue sparing followed by actual delivery of radiation and finally, post-treatment follow-up. AI systems are particularly suited to facilitate and improve the efficiency of this workflow. Machine learning has been proposed for automatic organ segmentation, error prevention, or treatment planning.^24,25^

#### Image acquisition

Imaging is an important part of radiation planning. As electronic density values have always been required for dose calculation algorithms, CT scans remain the basic imaging for planning purposes. However, this dependence on CT scans for generating electron density values for attenuation correction, is the reason why we have not been able to make a smooth transition from planning on a CT scan and diagnostic PET CT to MRI or PET MRI based planning, respectively; despite MRI having the advantage of better soft tissue delineation and ability of multiplanar image acquisition. For example, in treatment of a brain lesions, MRI, though a better imaging modality for tumour visualization can only be useful only when its scans are accurately mounted over a planning CT scan for better tumour delineation. As a solution to it, there have been constant efforts in the form of atlas-based method, sparse coding-based method, learning-based methods, to use the MRI data for generating a CT scan, also referred to as synthetic CT scans (sCT). Of the various methods to convert MRI data into sCT, deep embedding CNN, an AI based method, has emerged as more efficient, less time consuming, with generation of higher resolution images and less artefacts.^26^ So, in future, the use of AI may offset the need for a mandatory planning CT scan as synthetic CT scans can be generated from the MRI, quicker and with more reliable electron density data for plan generation. A synthetic CT scan will also have a positive impact in case of segmentation where the errors in fusion may be reduced as the sCT generated will get easily and more accurately fused with the MRI that forms the basis of the synthetic CT. Its use has been increasingly seen in MRI only prostate radiotherapy,^27,28^ where statistical decomposition algorithms have been utilized for sCT creation and plan generation from the primary MRI image.

#### Tumour and organs at risk segmentation

The contouring of OAR and target volumes is an important aspect of treatment planning in radiation oncology. This process, however, is time consuming and has marked interobserver variability depending on the skill level of the observer.^29^ Automatic contouring software have helped to speed up the process and improve consistency between observers. There are a number of commercially available products but these are not frequently used in clinical practice.^30^ In the recent past, a lot of effort has been put into learning new ways to recognize structures in a range of different imaging modalities (CT, PET, and MRI). Approaches range from knowledge-based algorithms such as atlas-based contouring, machine learning and statistical shape and appearance models; region-based methods such as adaptive thresholding, graph cuts and watershed contouring; or a combination of the knowledge- and region-based methods.^31^ Recently, machine learning techniques, and deep learning methods, using AI in particular, have become popular for a wider range of tasks. Tim Lustberg,^32^ in his recent study tried to compare the aspects of contouring manually, with auto-segmentation and with deep learning methods. The deep learning contouring outperformed the atlas-based contouring for lungs and spinal cord. Deep learning (DL) performed better for the oesophagus but further improvements remained necessary. When compared to manual methods performing a similar task, a median time saved of 79% was seen with the help of deep learning methods. Kuo Males and colleagues^33^ proposed a novel deep dilated CNN-based method for fast and consistent auto-segmentation of target and OARs volume delineation. They developed a novel multiple-scale convolutional architecture to extract multiple-scale context features, *e.g*. fine texture and boundaries and achieved pixelwise segmentation which are very useful for accurate auto-segmentation. A total of 218 patients chosen randomly were used for training, and the remaining 60 for validation. The dice similarity coefficient was used to measure segmentation accuracy and mean dice similarity coefficient values of deep dilated CNN were 87.7% for the CTV, 93.4% for the bladder, 92.1% for the left femoral head, 92.3% for the right femoral head, 65.3% for the intestine and 61.8% for the colon, which were better than the historically obtained values. Also, the test time was 45 s per patient for segmentation of all the CTV, bladder, left and right femoral heads, colon and intestine which is much faster to the time taken for conventionally drawing the structures. However, not all organs can be segmented with the same accuracy and consistency as there still are uncertainties associated with structures like optic chiasm and submandibular glands as shown in one study.^34^

With continuous ongoing research, we can expect, in near future for AI based methods like the CNN to have a significant role in generating the contours (including target volumes and OARs) for a patient, at a much faster and more consistent manner than what we are able to do at present.

#### Image registration

Image registration is the process of spatially aligning two or more image data sets of the same scene taken at different times, from different viewpoints. It takes the use of mathematical transformations applied to an image while making it more aligned to the reference image. There are various registration methods that are available in the market. In radiotherapy, the two major methods of registration used are the intensity-based techniques and rigid methods. In their survey about medical image registration techniques, Viergever et al reviewed the developments that took place between 1998 and 2016.^35^ They have stated, that deep learning approaches to image registration could very well be the new game changer in making the registration process easier and more user-friendly and have advocated applying deep learning concepts in making image registration an integral part of the entire spectrum of routine clinical imaging. Yang et al^36^ and Miao et al^37^ have used DL and CNN based methods respectively with faster, real-time registration than intensity-based models.

#### Radiation planning

Radiation planning is a complex process that involves using computer-based optimization for achieving specific dosimetric objectives before radiation is actually delivered. The process is currently laborious and time consuming and also involves some degree of “hit and trial”. AI can be used in getting a more refined and faster planning process. The different beam alignment and beam on time along with the complex dynamism of collimator movement has the potential to be optimized into an algorithmic scale and used effectively. McIntosh et al^38^ have pioneered the planning process of head and neck radiotherapy using a voxel-based dose prediction and dose mimicking method. Multiple patient atlas selection and machine learning methods were used to predict per voxel dose distributions which successfully integrated these methods with dose mimicking to create a novel fully automated radiotherapy treatment planning pipeline. Their AI-dependent methods have achieved the highest overall accuracy and in a fraction amount of time. Such strategy can be especially useful in adaptive radiotherapy planning where time is an important constraint.^39^ The adaptive radiotherapy planning process can be applied in real-time conditions, provided they are fast and accurate. In this case, the DL approaches for automatic segmentation and image registration, which are potentially faster than standard approaches, could allow reduction in radiation planning process. Machine learning is now used for knowledge-based planning that implies use of software tool to predict the dose–volume histogram of critical organs in relationship to the tumour. The tool helps to achieve quality inverse planning in less time.

Accessing and individualizing the dose constraints for a patient, during planning process, is another area where AI has been used.^40^ AI has the ability to affectively formulate algorithms which can include not only the conventional wisdom of dose–volume constraints, but also the various patient related factors like age, gender, ethnicity and genetic makeup into helping the clinician to make a better clinical assessment during that dose tradeoff in complicated radiation planning.^41^ Next step forwards the knowledge-based planning is being used experimentally for knowledge based adaptive planning where apart from inputs from radiology imaging information all relevant patient information, (clinical, dosimetry, tumour biology) will help in adapting radiotherapy to a personalized level thereby minimizing toxicity and improving tumour control at the same time. The clinical applications and usages will be a reality in near future.^42^ The future of AI is bright, in fact it has immense potentiality to predict which patients will benefit from radiation. PORTOS is the first of many future clinical radiogenomics assays which helps to determine the radiation sensitivity for tumour based on predictive biomarkers.^43^

#### Radiation delivery methods.

Monitoring intra- and interfraction motion during radiation has always been a challenging field, a near successful management of which has formed the basis of radical hypofractionated radiation. Monitoring of patient positioning and immobilization has tried to employ AI to reduce the uncertainty associated with motion. Ogunmolu et al^44,45^ have developed a soft-robot actuator for maskless H&N radiotherapy. Position-based visual-servoing of a radiotransparent soft robot was used to control the movements of flexion/extension of a manikin head. A Kinect RGB-D camera was used to measure head position and the error between the sensed and desired position was measured and used to control a pneumatic system which regulated pressure within an inflatable air bladder. Their results showed that the system was capable of controlling head motion to within 2 mm with respect to a reference trajectory.

The problems associated with tumour tracking have also been tackled with the help of AI. One limitation of tumour tracking is that, there exists a lag time of few microseconds between accessing the movement to finally correcting for it, calculated to be about 0.09 s in one study.^46^ This lag is usually corrected with the use predictive software which has an estimated accuracy of about 80%.^47^ Tumour tracking techniques can be refined with the incorporation of various patient data, especially breathing patterns and estimating the next breathing cycle.^48^ Park et al^49^ have proposed a new predictor for intra- and interfractional data variation, called intra- and interfraction fuzzy deep learning (FDL) which, equipped with data of breathing patterns, predicts the movements more accurately and decreases the computation time for tracking to be more accurate. They also found that the average computation time of interfraction FDL was 1.54 ms for both intra- and interfractional variation, which is much smaller than the existing methods. By accounting for the reduced intrafraction motion and the lag time between signal initiation and radiation delivery, significant reductions in treating volumes can be achieved with more confidence in the field of radiation oncology.

## Discussion

We have tried to summarize the prominent areas of upcoming association of AI with Oncology and Radiation Oncology (Table 1). The aim of the article was not so much as to detail the aspects of various AI associated techniques but to sensitize the reader about the various aspects of oncology that are and have potential to be affected with AI. As has been shown, citing above examples, it has the potential to affect almost all the fields of oncology by making sure that the vast data available with us with relation to the disease and patient can be effectively used to guide the clinicians. AI also has the ability to shorten the clinician’s and diagnostician’s time as well as effort to get a particular work done. The future holds great potential for applying AI to improve many aspects of the patient care process. In the coming times, AI can be utilized in more personalized treatments to effectively formulate an appropriate plan of diagnostic tests, treatment and follow up along with monitoring the patient population’s health and safety, leading to discovery of new medical knowledge that can directly impact the quality of care. There are other areas in oncology, like establishing guidelines and optimum frequency of follow up of a treated case, requirement of further biochemical tests or imaging and their frequency. Various governmental and non-governmental agencies can take the help of AI to cover for population-based needs like disease prevention methods, assisting in establishment of need based medical and oncology centres. With use of vast knowledge of demographic data, it can be made possible by AI to determine which areas need what form of investment, in regard to medical equipment or workforce. However, as with any new technology, come newer problems and challenges. Caution must be observed in making decision solely based on AI generated information as the main prerequisite for AI is the evaluation of data and a computer-based learning will always be limited in condition of paucity of data. There have been reports of manipulation of AI based methods as well. Methods to fool AI in the form of spoofing in the field of facial recognition have been well studied and documented.^50^ The deep division in technology among the developed and developing countries is also a factor that may cause judgemental errors.^51^ Data of developed countries cannot just be extrapolated to developing countries without expecting any discrepency. Hence, equity of data representation and keeping the geographical variation of diseases, population and health services in mind seems to be the way forward.

**Table 1.** Summary of the role of AI in radiation oncology

**Step of the workflow**	**Present AI role**	**Present and future implications**	**Reference no**.
**Image acquisition**	**Development of sCT scan from MRI images**.	No requirement of separate Planning CT Better for image registration	**26, 27 and 28**.
**Tumour segmentation**	**Deep learning methods in contouring OAR and target tissue**.	Faster, more consistent contouring Helpful in adaptive planning	**31, 32, 33 and 34**.
**Image registration**	**Deep learning approaches**.	**Faster and more precise image registration than intensity-based methods**	**36, 37**.
**Radiation planning**	**Voxel based dose prediction and dose monitoring**.	**Faster and more precise planning process**	**38 and 39**.
	**Using historical patients’ data and present patient’s characteristics**.	**Individualisation of dose constraints**	**40 41, 42 and 43**.
**Radiation delivery**	**Using soft resort activator controlling flexion of neck**.	**Decreased intra fraction motion**.	**44 and 45**.
	**Using deep learning for estimating breathing pattern**.	**Accurate tumour tracking with less errors of lag and predictive measures**	**48 and 49**.

It is the view of the authors, that the current emphasis should be given to acquire and access the large patient related data, especially from the developing countries, that can be used in deriving meaningful and applicable parameters for clinical use.

Lastly, it also should be borne in mind that a medical practitioner isn’t solely dependent on data, but on his experiences and judgements as well. His ultimate goal is to make patient’s life better keeping in mind his expectations, requirement and resources, factors which sometimes become more important than just quick and precision medicine

## Conclusion

AI generated algorithms and machine learning are on the path to becoming an important tool in the management decisions of radiation oncology. They have the potential to influence every step of the workflow right from screening, diagnosis, risk stratification, treatment planning, follow-up, and influence policy decisions. However, it should not be considered one stop solution or a magic wand for all the problems. Equity in data collection should be made an important step so that the developing countries may not be thrust upon the technology for which they are not well prepared . Lastly, the AI generated processes should be validated thoroughly before decisions are made solely on their algorithms and inferences.
